# Rab27b Regulates Mast Cell Granule Dynamics and Secretion

**DOI:** 10.1111/j.1600-0854.2007.00571.x

**Published:** 2007-05-04

**Authors:** Kouichi Mizuno, Tanya Tolmachova, Dmitry S Ushakov, Maryse Romao, Magnus Åbrink, Michael A Ferenczi, Graça Raposo, Miguel C Seabra

**Affiliations:** 1 Molecular and Cellular Medicine, National Heart and Lung Institute, Imperial College London SW7 2AZ UK; 2 Biological Nanoscience, National Heart and Lung Institute, Imperial College London SW7 2AZ UK; 3 Institut Curie, CNRS UMR144, Structure and Membrane Compartments, 75248 Paris France; 4 Department of Medical Biochemistry and Microbiology, Uppsala University, 751 05 Uppsala Sweden

**Keywords:** mast cell, organelle motility, Rab27, secretion

## Abstract

The Rab GTPase family regulates membrane domain organization and vesicular transport pathways. Recent studies indicate that one member of the family, Rab27a, regulates transport of lysosome-related organelles in specialized cells, such as melanosomes and lytic granules. Very little is known about the related isoform, Rab27b. Here we used genetically modified mice to study the involvement of the Rab27 proteins in mast cells, which play key roles in allergic responses. Both Rab27a and Rab27b isoforms are expressed in bone marrow-derived mast cells (BMMC) and localize to secretory granules. Nevertheless, secretory defects as measured by β-hexosaminidase release *in vitro* and passive cutaneous anaphylaxis *in vivo* were found only in Rab27b and double Rab27 knockout (KO) mice. Immunofluorescence studies suggest that a subset of Rab27b and double Rab27-deficient BMMCs exhibit mild clustering of granules. Quantitative analysis of live-cell time-lapse imaging revealed that BMMCs derived from double Rab27 KO mice showed almost 10-fold increase in granules exhibiting fast movement (>1.5 μm/s), which could be disrupted by nocodazole. These results suggest that Rab27 proteins, particularly Rab27b, play a crucial role in mast cell degranulation and that their action regulates the transition from microtubule to actin-based motility.

Mast cells play central roles in allergic and T-helper lymphocyte type 2-mediated immune responses (1). Mast cell secretory granules store various inflammatory mediators including histamine and serotonin (2,3), and the activation of the high-affinity immunoglobulin E (IgE) receptor induces exocytosis of the granules, resulting in inflammatory mediators release (4,5). The study of mast cell secretion is thus of great relevance to therapeutically modulate the activity of mast cells in the control of allergic diseases, such as asthma.

The Rab family of Ras-like GTPases, which comprise more than 60 members in mammalian cells, have been implicated in regulated secretion (6). Rab3 proteins have been studied for many years in neuronal and non-neuronal regulated exocytotic events, but the precise mechanism by which they operate remains controversial (7–9). More recently, Rab27a has been implicated as a positive regulator of regulated exocytosis in cytotoxic T lymphocytes (10,11), pancreatic β-cells (12,13), neuroendocrine (14), platelets (15) and prostate cells (16). Rab27a appears to promote the switch between microtubule (MT)-based organelle motility to actin-based motility by recruiting myosin-interacting proteins, such as Melanophilin and Myrip. These proteins in turn bind to unconventional myosins such as Myosin Va, thereby promoting tethering and movement along the actin cytoskeleton (17,18). Mutations in the human *RAB27A* gene cause Griscelli syndrome. This disorder is characterized by partial albinism of the skin and the hair associated with variable immunodeficiency, and caused by motility defects in melanosomes and lytic granules (18–21). Furthermore, Rab27a recruits effectors of the synaptotagmin-like protein family (such as granuphilin) and Munc13-4, which may act downstream of myosin in the docking of secretory vesicles with the plasma membrane (22,23). The present evidence thus suggests that Rab27a acts as a positive regulator of regulated secretion in either conventional Golgi-derived granules in exocrine and endocrine cells, or lysosome-related organelles in hematopoietic cells and melanocytes (19,21,24).

The mast cell secretory granules are considered lysosome-related organelles as they share features with lysosomes, such as containing lysosomal proteins (2,21). Rab3d was proposed to be a regulator of mast cell secretory granule exocytosis, because overexpression of Rab3d results in a reduction of exocytosis in the model cell line, RBL-2H3 (25). However, mast cell secretion in Rab3d knockout (KO) mice is unaffected (26). More recently, Rab27a and its effector Munc13-4 were implicated in secretory granule exocytosis in RBL-2H3 cells (27,28).

The Rab27 subfamily consists of two isoforms sharing 71% amino acid sequence similarity, Rab27a and Rab27b (29–31). Rab27a is widely expressed (24), whereas the expression of Rab27b is more restricted and detectable in platelets, the pituitary gland and the digestive tract (31–33). Very little is known about the function of Rab27b other than being implicated as a positive regulator of exocrine gland exocytosis, namely, of zymogen granules in pancreatic acinar cells (34) and amylase granules in parotid acinar cells (35). We have recently generated a Rab27b KO using a conditional Cre–loxP strategy, which enabled analysis of Rab27 functions in specialized cells *in vivo* and *in vitro* (36). We have shown that *Rab27b* KO and double *Rab27a/Rab27b* KO mice exhibit significant hemorrhagic disease. Platelets exhibited impaired *in vitro* aggregation properties, and reduced number and secretion of dense granules (36). In this paper, we describe mast cell function studies using Rab27a KO, Rab27b KO and double KO mice. We show surprisingly that despite expression of both isoforms, mast cells exhibit opposite phenotypes in individual KOs. Rab27b appears to be the critical regulator of mast cell degranulation by enabling peripheral actin-mediated retention of granules near the plasma membrane prior to exocytosis.

## Results

**Figure 1:**
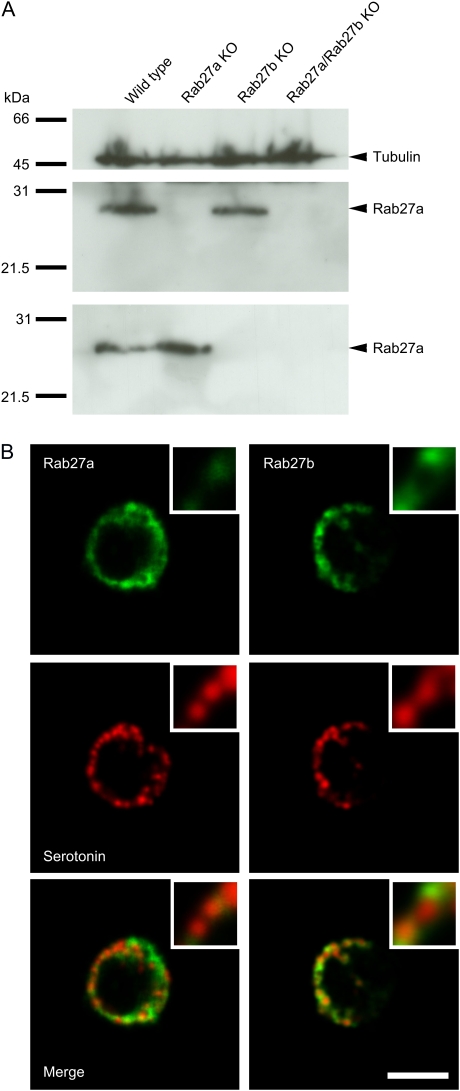
Rab27 proteins in BMMCs A) Bone marrow-derived mast cells derived from C57BL/6J wild-type, Rab27a KO (*Rab27a^ash/ash^*), Rab27b KO (*Rab27b*^−/−^) and double KO (*Rab27a^ash/ash^Rab27b*^−/−^) mice were subjected to immunoblot analysis as described under *Materials and Methods*. B) Bone marrow-derived mast cells derived from C57BL/6J wild-type mice were subjected to immunofluorescence using anti-Rab27a, anti-Rab27b and anti-serotonin antibodies. Representative single midsection images by confocal microscopy are shown. Inserted images are enlarged. Bar, 10 μm.

To determine the expression of Rab27 isoforms in mast cells, we prepared interleukin-3 (IL-3) and stem cell factor-dependent bone marrow-derived mast cells (BMMC) from Rab27a KO or more precisely the naturally occurring *ashen* (*ash*) strain, *Rab27a^ash/ash^*, the recently created Rab27b KO (*Rab27b*^−/−^)(36), the double Rab27 KO resulting from the cross of these two strains (*Rab27a^ash/ash^*; *Rab27b*^−/−^) mice and respective heterozygote control littermates. All BMMCs studied expressed both c-Kit and high-affinity IgE receptor, markers that are characteristic of mature BMMCs (data not shown). We detected expression of both Rab27a and Rab27b proteins using isoform-specific antibodies in wild-type BMMC lysates (Figure 1A). The absence of reactivity in the respective KO lines confirms that these lines indeed are defective in the expression of the respective Rab27 isoform. We conclude that both Rab27a and Rab27b proteins are expressed in BMMCs and that their expression appears to be dispensable for the cytokine-dependent emergence and differentiation of BMMC.

Next, we determined the intracellular localization of Rab27a and Rab27b in BMMCs using immunofluorescence. Immunostaining with the anti-Rab27b antibody indicated that Rab27b exhibited a punctate vesicular staining pattern throughout the cytoplasm, which overlapped with the signal obtained from the anti-serotonin antibody (Figure 1B). The Rab27a immunostaining pattern was more diffuse, although some discrete punctate staining was also observed (Figure 1B). These results suggest that Rab27b and to a lesser extent Rab27a are associated with serotonin-containing mast cell secretory granules.

**Figure 2:**
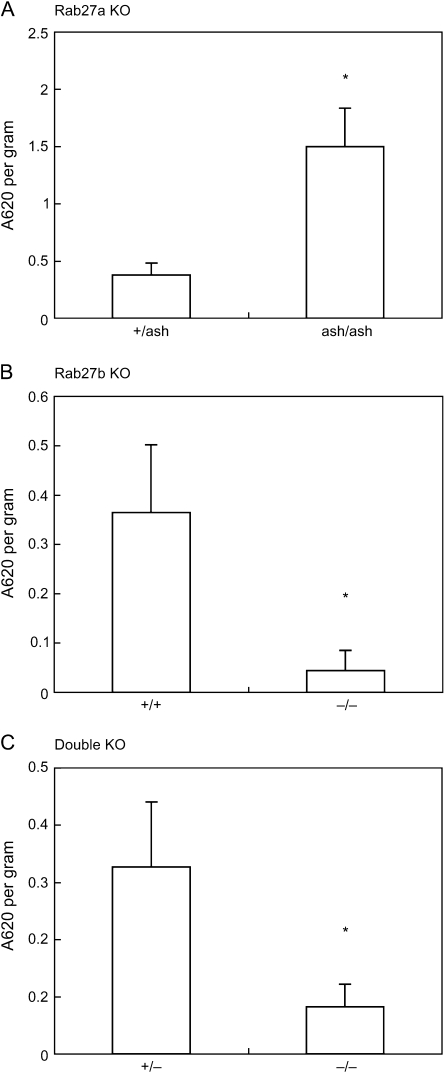
>Passive cutaneous anaphylaxis assays in Rab27-deficient mice *Rab27a^ash/ash^*(ash/ash) or heterozygote littermate (ash/+) mice (A), *Rab27b*^−/−^ (−/−) or heterozygote littermate (−/+) mice (B), and *Rab27a^ash/ash^Rab27b*^−/−^ (−/−) or heterozygote littermate (−/+) mice (C) were subjected to PCA assays as described under *Materials and Methods*. Data presented are the mean with standard error of the mean of at least nine independent experiments. *p < 0.05 (unpaired Student's *t*-test).

The presence of the Rab27 isoforms in BMMCs raised the possibility that they could be controlling regulated secretion in these cells and thus allergic responses in mice. As a first test, we assessed the ability of the single and double Rab27 KO lines to react against immune challenge using the passive cutaneous anaphylaxis (PCA) response because anaphylaxis mediated via high-affinity IgE receptor is regarded predominantly as a mast cell-dependent event (4). Mice were challenged intradermally with IgE or saline in different locations of the dorsal dermis. After sensitization, mice were injected intravenously with antigen and dye, and PCA responses were measured after 1 h. A marked reduction in PCA response was observed in Rab27b and double KO mice (Figure 2B,C), despite the presence of toluidine-positive cells in the dorsal dermis (Figure S1). Surprisingly, the PCA response was enhanced in Rab27a KO mice dermis (Figure 2A). These differences were statistically significant (p < 0.05).

**Figure 3:**
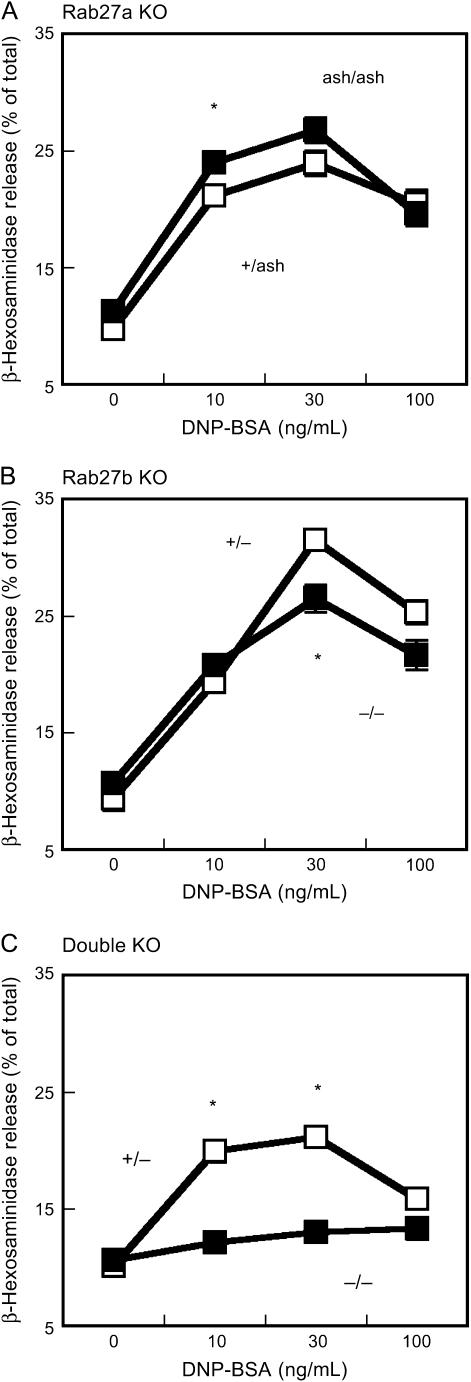
Antigen-induced β-hexosaminidase release in BMMCs derived from Rab27-deficient mice Bone marrow-derived mast cells derived from *Rab27a^ash/ash^* (ash/ash) or heterozygote littermate (ash/+) mice (A), *Rab27b*^−/−^ (−/−) or heterozygote littermate (−/+) mice (B) and *Rab27a^ash/ash^Rab27b*^−/−^ (−/−) or heterozygote littermate (−/+) mice (C) were subjected to β-hexosaminidase release assay as described under *Materials and Methods*. After sensitization with anti-dinitrophenyl IgE, the cells were stimulated with various concentration of DNP–BSA for 20 min. Each point was determined in triplicate and presented as mean with standard error of the mean. The experiments shown are representative of at least three independent experiments. *p < 0.05 (Mann–Whitney test).

The results above suggested that both Rab27a and Rab27b expression was required for normal mast cell function, presumably in the control of regulated secretion. Thus, we next examined the effects of Rab27 KOs on antigen-induced β-hexosaminidase release from BMMC *in vitro*. These *in vitro* assays are rather variable from experiment to experiment as they depend on the culture conditions and can only be compared within experiments. Furthermore, the experiments shown are only qualitatively representative as the magnitude of the response and the differences observed also varied in independent experiments. The antigen, dinitrophenyl (DNP)-BSA, induced β-hexosaminidase release from BMMC cells sensitized with anti-DNP IgE (Figure 3). In Rab27b KO BMMCs, we observed a small decreased in the secretory response (Figure 3B). In Rab27a KO BMMCs, we observed a small increase in stimulated secretion (Figure 3A). Strikingly, β-hexosaminidase release was more consistently reduced in double Rab27 KO BMMCs (Figure 3C). The general trend of these experiments was confirmed in antigen-induced histamine release assays (data not shown). These results were consistent with the PCA assay *in vivo* and suggested that Rab27 proteins are key regulators of antigen-induced secretion in mast cells.

**Figure 4:**
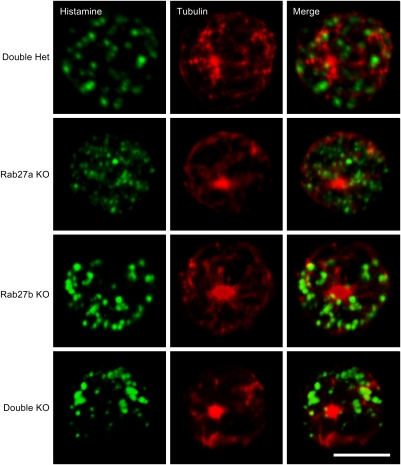
Immunofluorescence analysis of mast cell secretory granules in BMMCs derived from Rab27-deficient mice Bone marrow-derived mast cells derived from *Rab27a*^*ash*/+^*Rab27b*^−/+^ (Double het), *Rab27a^ash/ash^* (Rab27a KO), *Rab27b*^−/−^ (Rab27b KO) and *Rab27a^ash/ash^Rab27b*^−/−^ (Double KO) were subjected to immunofluorescence as described under *Materials and Methods*, and stained with anti-histamine (green) and anti-alpha-tubulin (red) antibodies. Representative projected images using confocal microscopy as described under *Materials and Methods* are shown. Bar, 5 μm.

Recent studies on Rab27a in melanocytes have led to the proposal that the GTPase promotes the switch between MT-based motility to actin-based motility of melanosomes through the recruitment of Melanophilin and Myosin Va (17,18). We therefore studied whether mast cell granule localization was affected in the KO lines. Using immunofluorescence of fixed cells, we observed scattered histamine-positive granules (Figure 4) or serotonin-positive granules (data not shown) throughout the cytoplasm in wild-type and Rab27a KO-derived BMMCs. Conversely, in Rab27b KO or double KO BMMCs, the histamine-positive granules appeared more clustered (Figure 4) in a subset of BMMCs, more specifically 32% of Rab27b KO (*n* = 34) and 63% of double KO (*n* = 8) BMMCs, compared with no obvious accumulation of granules in wild-type (*n* = 55), *Rab27a^+/ash^* (*n* = 11), *Rab27a^ash/ash^* (*n* = 36), *Rab27b*^+/−^ (*n* = 21) and *Rab27a*^+/*ash*^; *Rab27b*^+/−^ (*n* = 8) BMMCs.

**Figure 5:**
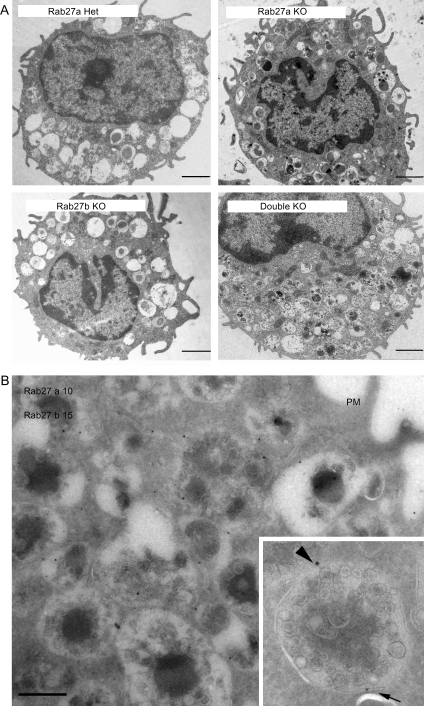
Electron microscopy analysis of BMMCs A) Ultrathin sections of epon-embedded BMMCs from the indicated strains. Bar, 2 μm. B) Ultrathin cryosections of BMMCs from EGFP-Rab27a mice were double immunogold labeled for Rab27b (PAG15) and Rab27a (PAG10). Rab27a was detected with anti-GFP. Labeling for both Rab27b (arrowheads) and Rab27a (arrows) could be detected on the same granules Bar, 500 nm. Inset: example of a type II granule containing an electron-dense core and small internal vesicles. Bar, 250 nm. PM, plasma membrane.

To extend the above results and to examine granule morphology in detail, we performed electron microscopy. All BMMCs analyzed exhibited type I (multivesicular), type II (multivesicular with dense core) and type III (dense core) granules (2) without obvious differences between samples (Figure 5A). Using immunoelectron microscopy, we observed labeling for both Rab27a and Rab27b at the limiting membrane of mast cell secretory granules of all types and the relative distribution of both proteins appeared similar (Figure 5B), although Rab27a staining appeared more sparse.

**Figure 6:**
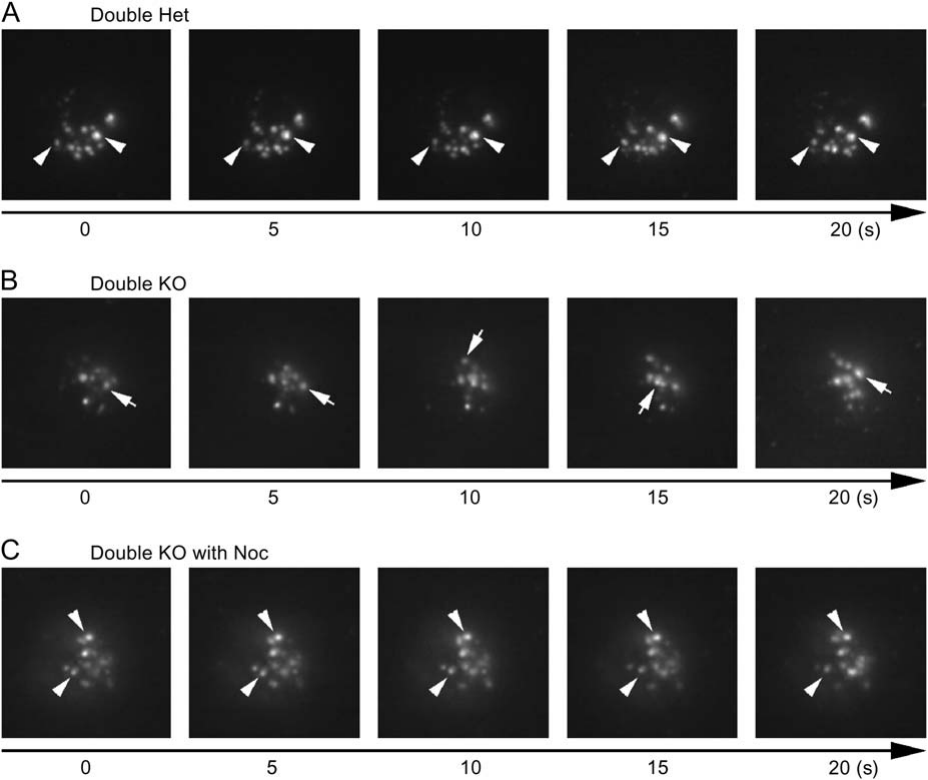
Secretory granule dynamics in BMMCs Bone marrow-derived mast cells derived from *Rab27a*^*ash*/+^*Rab27b*^−/+^ (Double het) (A), or *Rab27a^ash/ash^Rab27b*^−/−^ (Double KO) mice in the absence (B) or presence of nocodazole (C) were labeled with LysoTracker Red DND-99 and subjected to TIRF microscopy as described under *Materials and Methods*. Arrowheads point to granules that remain still and arrows to granules that exhibit fast movement (<1.5 μm/s, see also Table 1). Selected frames from time-lapse recordings are presented. Bar, 2.5 μm.

We then studied mast cell secretory granule dynamics by total internal reflection fluorescence (TIRF) microscopy. Total internal reflection fluorescence microscopy allows real-time imaging of fluorescence near the plasma membrane. We used LysoTracker Red DND-99 as a marker for secretory granules as we observed its concentration in serotonin-positive granules (data not shown). In general, we observed that the vast majority of granules in BMMCs was stationary and a minority of granules was motile (Figure 6; Movie S1). However, quantitative analysis revealed significant differences between strains. Granules exhibiting displacements over 1.5 μm were increased by almost 10-fold in double KO BMMCs (and Rab27b KO BMMCs, data not shown) as compared to double heterozygote controls (Table 1; Movie S2). Conversely, the percentage of motile granules defined as those with displacements exceeding 0.5 μm and average velocity exceeding 0.1 μm/s decreased in double KO BMMCs (Table 1). Therefore, and given the large increase of fast-moving granules (>1.5 μm/s) in double KO cells, the twofold decrease in motile granules can be accounted by a decrease in slow moving granules (<1.5 μm/s displacement). We hypothesized that the fast movements observed were attributable to MT-based motility; so we treated cells with nocodazole, a MT-disrupting agent. Consistently, nocodazole treatment inhibited the fast and long-range movements of LysoTracker-labeled granules in double KO BMMCs (Figure 6C; Movie S3). These results suggest that the absence of Rab27 proteins leads to more prominent MT-based motility of mast cell granules.

**Table 1:** Analysis of LysoTracker-positive granule movement in BMMCs

Genotype	Average velocity (μm/s)	Motile granules(%)	Displacement (μm)			
			
			Number studied	0.5–1.0	1.0–1.5	>1.5
Double heterozygote	0.16 ± 0.06 (*n* = 408)	21.3	87	90.8%	8.0%	1.2%
Double KO	0.15 ± 0.07 (*n* = 521)	12.7	66	78.8%	10.6%	10.6%
Double KO + nocodazole	0.05 ± 0.03	2.7	ND	ND	ND	ND

ND, not determined. The LysoTracker-positive granule movements were analyzed as described under *Materials and Methods*. Motile granules are defined as ones with displacement exceeding 0.5 μm and average velocity exceeding 0.1 μm per second.

## Discussion

The present studies suggest a key role for Rab27 proteins in the release of secretory granules in mast cells. Our data indicate that simultaneous loss of both Rab27a and Rab27b leads to a severe secretory defect *in vitro* and poor reactivity *in vivo*. The granule dynamics are affected in double KO mice with a dramatic increase in fast-moving granules, suggesting that the underlying defect could be impaired transition between MT-based and actin-based motility prior to exocytosis. Single KO of Rab27 isoforms further suggests that Rab27b may be the key regulator in mast cells given that the Rab27b phenotype mimics, albeit less dramatically, that of the double KO.

The mode of action of Rab27a in melanocytes as a key regulator of melanosome motility to recruit myosin motors to mature melanosomes and thus induce their peripheral retention has been intensively investigated (17,18). Our present studies suggest that a similar mechanism may be operating in mast cell granule dynamics. Rab27 isoforms appear to be required for the transition from MTs to actin, as revealed by our live-cell imaging and quantitative analysis (Figure 6; Table 1; Movies S1, S2 and S3). Our data suggest that MT-dependent secretory granule movements are increased in double Rab27 KO mast cells, presumably because of a disconnection between MT and actin motor systems (Figure 6; Table 1). In Rab27b KO mast cell, a similar but more subtle phenotype is observed, both morphologically with some cells showing evidence of perinuclear granule clustering (Figure 4) and functionally with increased numbers of fast-moving granules (data not shown). Therefore, our data is consistent with the involvement of Rab27 proteins, particularly Rab27b in MT-actin transition as previously established for Rab27a in skin melanocytes. We speculate that Rab27 proteins recruit a Rab-and-Myosin linker effector such as Melanophilin or Myrip, which will then recruit an unconventional myosin. Further studies should aim at identifying the Rab27 molecular partners in mast cell granule dynamics.

Mast cells represent a cell type where both Rab27a and Rab27b are expressed, providing an opportunity to determine the relative contributions of each isoform and address redundancy issues. Our studies suggest that when Rab27b is absent, then Rab27a may partially compensate for its loss as a positive regulator of exocytosis, given that the secretory defect in the double KO cells is more severe than in the single *Rab27b* KO. The partial redundancy of the Rab27 isoforms was expected given our previous studies on the pathogenesis of Griscelli syndrome where we showed that transgenic heterologous expression of Rab27b in melanocytes could compensate for melanosome clustering and coat color dilution in *Rab27a* KO (*ash*) mice (32). So far, only a few cases of functional redundancy have been reported for the Rab family, namely the requirement for simultaneous KO of all four Rab3 isoforms to achieve lethality (37) and the redundant role of Rab38 and Rab32 in melanosome biogenesis in skin melanocytes (38).

One surprising result obtained here was the increased secretory responses in BMMCs derived from *Rab27a^ash/ash^* mice, raising the possibility that Rab27a and Rab27b might have distinct roles in mast cell secretion. A possible interpretation is that Rab27a performs an inhibitory function on regulated exocytosis in mast cells. Interestingly, the secretory actions attributed to the related Rab3 proteins in the neuroendocrine PC12 cell line are also inhibitory. Overexpression of any Rab3 isoform leads to a strong decrease in Ca^++^-regulated exocytosis (39,40). However, more detailed analysis revealed that the inhibitory effect on regulated exocytosis is indirect and results from an activation of constitutive exocytosis (40). Therefore, one interesting possibility is that Rab27a may be performing a similar action in mast cells. It is noteworthy that Rab27 and Rab3 are able to interact with the same effectors, such as Rabphilin, Granuphilin and Noc2 (23).

The findings presented here highlight some of the limitations of studying cultured cell lines and conversely the importance of studying primary cells in specialized systems of medical relevance. A previous study using RBL-2H3 cells, a rat basophilic leukemia-derived cell line commonly used as a model for mast cells implicated Rab3d in regulated exocytosis (25). However, Rab3d KO mice exhibit normal mast cell responses (26). Recent studies suggested that Rab27a and its effector Munc13-4 regulate exocytosis of secretory granules in the same cell line (27,28). Our studies with primary cells suggest instead that Rab27a performs an inhibitory role.

The identification of severely reduced mast cell responses *in vivo* in the *Rab27b* KO mouse is the first report that Rab27b contributes to allergic responses in mammals and suggests that Rab27b may be a future drug target for allergic diseases, such as asthma. In fact, the restricted pattern of expression observed for Rab27b compared with Rab27a further strengthens this possibility, suggesting that interfering with Rab27b function may lead to rather specific effects. Future studies should assess the therapeutic potential of interfering with Rab27b function.

## Materials and Methods

### Antibodies and reagents

The primary antibodies, mouse monoclonal anti-Rab27a antibody, rabbit polyclonal anti-Rab27a antibody and rabbit polyclonal anti-Rab27b antibody were as described previously (32). Mouse anti-serotonin antibody was purchased from Biogenesis (Pool, UK). Rabbit anti-histamine antibody was purchased from Research Diagnostics, Inc. (Flanders, NJ, USA). Mouse anti-alpha-tubulin DM 1A antibody was purchased from Sigma (St Louis, MO, USA). LysoTracker Red DND-99, Rhodamine-phalloidin, Alexa488- and Alexa568-conjugated secondary antibodies were purchased from Molecular Probes (Eugene, OR, USA). Horseradish peroxidase-linked antibodies were purchased from DAKO (Glostrup, Denmark). DNP–BSA was prepared as previously described (41).

### Generation of Rab27b KO and double Rab27 KO mice

All mice were bred and maintained under UK project license PPL 70/6176 at the Central Biomedical Services of Imperial College, London, UK. C57BL/6J wild-type mice were purchased from B&K Universal Limited (Hull, UK). *ashen* mice (C57BL/6J-*ash*/*ash*) (32) and EGFP-Rab27a transgenic mice (24) were described previously. The generation of Rab27a KO mice and double Rab27 KO was described elsewhere (36).

### Primary mast cell culture

Bone marrow was obtained from the femur of mice of various genetic background and cultured at 37°C/5% CO_2_, in RPMI1640 (Invitrogen Life Technologies, Paisley, UK) supplemented with 10% heat-inactivated fetal calf serum, 55 μm 2-mercaptoethanol (2-ME) (Sigma-Aldrich, Poole, UK), 10 U/mL penicillin/streptomycin, 20 ng/mL recombinant murine IL-3 and 20 ng/mL recombinant murine SCF (Peprotech, London, UK). The medium was changed every 3–4 days and non-adherent cells were transferred to new culture flasks. Bone marrow-derived mast cells appeared in the culture after 2 weeks and to determine the expression of high-affinity IgE receptor, cell aliquots were first exposed to 2.4G2 rat anti-mouse FcgammaRII/RIII antibody (BD Biosciences, San Diego, CA) and subsequently incubated with SPE-7 mouse IgE (Sigma-Aldrich). Cell staining was performed with fluorescein isothiocyanate-labeled monoclonal rat anti-mouse IgE antibody (BD Biosciences) and phycoerythrin-labeled rat anti-c-Kit antibody (BD Biosciences). As analyzed with a FACScalibur system (BD Biosciences), >98% of the cells expressed both high-affinity IgE receptor and c-Kit after 4 weeks of differentiation. The experiments described below were all performed with 4- to 9-week-old mast cells.

### Immunoblotting

Bone marrow-derived mast cells were washed twice in PBS and solubilized in 2× SDS sample buffer. Samples were loaded on polyacrylamide gels and transferred to polyvinylidene difluoride membranes. After blocking, the membranes were treated with the anti-Rab27a or anti-Rab27b antibodies and immunoreactive proteins were detected using the enhanced chemiluminescence protocol (Amersham Biosciences, Piscataway, NJ, USA) with horseradish peroxidase-linked secondary antibody (DAKO).

### Passive cutaneous anaphylaxis

Mice were anesthetised and injected intradermally at two dorsal sites (left and right flank) with 100 ng of murine monoclonal anti-DNP IgE (SPE-7; Sigma) in 20 μL of saline. Twenty-four hours later, the mice were injected intravenously with 100 μg of DNP–human serum albumin in 100 μL 0.5% Evan's blue dye (added to permit visual localization of increased vascular permeability) in saline. Thirty minutes later, the mice were killed by exsanguination under anesthesia, tissue sections around the intradermal injection site excised and weighted, followed by extraction of extravasated Evan’s blue dye by incubation of biopsies in 1 mL formamide at 55°C for 24 h and spectophotometric measurement of absorbance at 620 nm (A620). Passive cutaneous anaphylaxis response was quantified by dye extraction from IgE-injected and saline-injected skins. Data are expressed as A620 of IgE-sensitized area minus A620 of saline-injected area per weight of sample (g).

### Histochemistry

Specimens of dorsal dermis were surgically excised and processed for histochemistry using standard procedures (24). Paraffin sections (4 μm) were stained with toluidine blue and eosin as counter staining.

### Measurement of β-hexosaminidase release

Bone marrow-derived mast cells were washed twice in HEPES buffer [140 mm NaCl, 5 mm KCl, 0.6 mm MgCl_2_, 1 mm CaCl_2_, 10 mm HEPES (pH 7.4) and 5.5 mm glucose] and sensitized for 1 h by 2 μg/mL anti-DNP mouse monoclonal IgE (Sigma-Aldrich). After sensitization, the cells were washed twice with HEPES buffer, stimulated with DNP–BSA for 20 min at 37°C, and the reaction stopped by addition of ice-cold HEPES buffer. After centrifugation (3000 ×***g***, 4°C), supernatants were collected and supplemented with 10% Triton-X-100. Cell pellets were lysed with 10% Triton-X-100 (in distilled water) and supplemented with ice-cold HEPES buffer. Aliquots (100 μL) of supernatants or cell lysates were incubated with 250 μL of 1.3 mg/mL *p*-nitrophenyl-*N*-acetyl-β-d-glucosamine (enzyme substrate) dissolved in 0.1 m sodium citrate (pH 4.3) for 90 min at 37°C. Then 750 μL of a 0.2 m glycine (pH 10.5) was added, and absorbance was measured at 405 nm. Total enzyme activity was calculated as the sum of supernatant and cell pellet activities. β-hexosaminidase release was expressed as the percentage of total enzyme content.

### Assay of histamine release

Bone marrow-derived mast cells were washed twice in HEPES buffer and sensitized for 1 h by 2 μg/mL anti-DNP mouse monoclonal IgE. After sensitization, the cells were washed twice with HEPES buffer and stimulated with DNP–BSA. After stimulation for 20 min at 37°C, the reaction was terminated by addition of ice-cold HEPES buffer. After centrifugation (3000 × ***g***, 4°C), supernatants were harvested and perchloric acid was added. The histamine content was measured by the *o*-phthalaldehyde fluorometric procedure (42). Briefly, the medium (0.6 mL) was transferred to a tube containing 0.6 mL of 3% HClO_4_, and cells in each well were used to measure histamine remaining in the cells by adding 1.2 mL of 1.5% HClO_4_. To 0.5 mL of sample, a mixture of 100 μL of 3 N KOH, 0.3 g of NaCl and 630 μL of *n*-butanol was added. Then, the samples were centrifuged at 1000 × ***g*** for 3 min. The upper organic phase (500 μL) was transferred to tubes containing 300 μL HCl (0.2 N) and 950 μL *n*-heptane. To the 250 μL of the lower aqueous phase, 100 μL of NaOH (1 N) was added. The histamine–OPT reaction was carried out by incubation with 0.1 mL of OPT (0.05% in methanol) for 40 min on ice, and terminated by addition of 100 μL citrate (2 m). The fluorescence of the sample was measured at 450 nm emission excited at 360 nm with a LS 50B (Perkin). Histamine release was expressed as the percentage of total histamine content.

### Confocal fluorescence microscopy

Bone marrow-derived mast cell from various genetic backgrounds were plated on poly-lysine-coated cover glass. After 30 min, the cells were fixed with 3.7% formaldehyde in PBS for 20 min at room temperature. The cells were washed three times with PBS and were treated with 0.1% Triton-X-100 in PBS for 15 min at room temperature. After blocking, the cells were incubated with the first antibodies for 2 h at room temperature and were washed three times with PBS, followed by incubation with the Alexa488- or Alexa568-conjugated secondary antibody for 1 h at room temperature. The cells were washed three times with PBS and were mounted on slide glasses using DAKO mounting solution (DAKO). Fluorescence was visualized through a Leica TCSNT confocal system in conjunction with Leica DM IRBE. Z-axial sections were collected about 0.6-μm step and the projected images were represented. Images were filtered using imagej and fftj and deconvolutionj plugin (http://rsb.info.nih.gov/ij/).

### Total internal reflection fluorescence microscopy

For labeling of mast cell secretory granules, BMMC was incubated with 200 nm LysoTracker Red DND-99 (Molecular Probes) for 30 min at 37°C. After labeling, the cells were washed twice with HEPES buffer plus 55 μm 2-ME. Fluorescence imaging was performed using Zeiss Axiovert 200 inverted microscope modified for objective-type TIFR microscopy (Till Photonics, Munich, Germany). An argon laser 514 nm line was used to excite LysoTracker Red DND-99 through a 100 × 1.45 NA Zeiss Planofluor objective. During observation, the cells were kept on MS2000 stage (Applied Scientific Instrumentation, Eugene, OR) equipped with a Focht chamber system (FCS2, Biotechs, Butler, PA) at 30°C. The images were recorded using a PCO SensiCam CCD camera and analyzed using imagej software. The images of each cell were recorded with 750 ms exposure at 1-s intervals. The scale is 16 pixels per micrometer. The image size was 236 × 236 pixels.

### Quantitative analysis of granule dynamics

Total internal reflection fluorescence microscopy image sequences were analyzed using volocity quantitation software (Improvision, Coventry, UK). Fluorescent particles were first selected by intensity threshold and size, then smoothed by Gaussian filter and tracked using the shortest path algorithm.

### Electron microscopy

Bone marrow-derived mast cells on coverslips were fixed with 1.25% glutaraldehyde in 0.1 m cacodylate buffer for 24 h. After several washes with 0.1 m cacodylate buffer, the cells were postfixed with 2% OsO_4_, dehydrated in ethanol and embedded in epon while on the coverslips. Ultrathin sections were prepared and counterstained with uranyl acetate and lead citrate before observation. For immunogold labeling, mast cells were fixed with 2% (w/v) paraformaldehyde or with a mixture of 2% PFA and 0.2% glutaraldehyde in 0.1 M phosphate buffer, pH 7.4. Bone marrow-derived mast cell from EGFP-Rab27a mice were processed for ultracryomicrotomy as described (2). Ultrathin sections were prepared with an Ultracut FCS ultracryomicrotome (Leica, Wetzlar, Germany), and double immunogold labeled with antibodies and protein A coupled to 5 or 10 nm gold. Rab27b was detected with a specific antibody and Rab27a with an anti-GFP antibody. Sections were observed and photographed under a Philips CM120 Electron Microscope (FEI Company, Hillsboro, OR). Digital acquisitions were made with a Keen View numeric camera (SIS, Klausdorf, Germany).

## Supplementary Materials

Figure S1 Toluidine blue staining of skin sections Dermal mast cells were detected with toluidine blue staining in double heterozygote, Rab27a KO, Rab27b KO and double KO mice back dermis. The sections were also stained with eosin. The highly metachromatic cells are mast cells. Arrowhead, mast cell. Bar, 200 μm.

Movies S1, S2, and S3 Secretory granule dynamics in BMMCs Images were captured every second. Playback speed is 7 frames per second. Movie S1: Double Het; Movie S2: Double KO; Movie S3: Double KO with nocadazole. See Figure 6 for more details.
